# Advanced Gouty Nephropathy Complicated With Type 1 Renal Tubular Acidosis: A Case Report

**DOI:** 10.7759/cureus.71094

**Published:** 2024-10-08

**Authors:** Chihiro Uda, Ryuichi Ohta, Chiaki Sano

**Affiliations:** 1 Family Medicine, Fuchu Hospital, Osaka, JPN; 2 Community Care, Unnan City Hospital, Unnan, JPN; 3 Community Medicine Management, Shimane University Faculty of Medicine, Izumo, JPN

**Keywords:** chronic kidney disease, family medicine, general medicine, gouty nephropathy, hyperuricemia, hypokalemia, metabolic acidosis, renal tubular acidosis

## Abstract

This report describes the case of a 53-year-old woman with chronic kidney disease (CKD) exacerbated by a gout flare who presented with renal tubular acidosis (RTA), hypokalemia, and hyperuricemia. Despite outpatient management for gouty nephropathy, the patient experienced progressive hypokalemia, leading to hospitalization. Upon admission, she was diagnosed with type 1 RTA, characterized by metabolic acidosis and severe hypokalemia, refractory to initial potassium supplementation. The patient's medical history included gout, chronic renal failure, and other comorbidities, complicating her condition. Treatment included aggressive potassium replacement and ongoing management of her gout and CKD. Over several hospital days, her potassium levels stabilized, and she was discharged on oral potassium supplements. This case emphasizes the importance of monitoring electrolyte imbalances and managing uric acid levels in patients with chronic gout and kidney disease to prevent complications such as RTA. Comprehensive management strategies, including dietary and pharmacological interventions, are critical to prevent the progression of gouty nephropathy and improve patient outcomes.

## Introduction

Renal tubular acidosis (RTA) is a disorder characterized by normal anion gap metabolic acidosis, where acid secretion in the renal collecting ducts or bicarbonate reabsorption in the proximal tubules is impaired, despite the absence or mild presence of glomerular dysfunction [1]. RTA is categorized into type I, type II, and type IV, each having hereditary and secondary forms [2]. In the collecting ducts, non-volatile acids accumulated in the body are excreted into the lumen via H+-ATPase. In type I RTA, impaired H+ excretion in the collecting ducts leads to difficulty in acidifying the urine, making it challenging to correct the acidosis [3]. The molecules responsible for H+ excretion in the collecting ducts include H+-ATPase on the luminal membrane and anion exchanger one on the basolateral membrane [3]. The former is implicated in autosomal recessive type I RTA, whereas the latter is associated with autosomal dominant type I RTA [4]. In type I RTA, hydroxyapatite in bones is used to neutralize the accumulated acid due to impaired excretion, which leads to increased bone resorption if left untreated [5]. Conditions such as rickets, osteomalacia, and increased urinary calcium excretion may develop [3]. Citrate reabsorption in the proximal tubules is also increased to maintain the balance of bases. As a result, hypercalciuria and hypocitraturia occur, leading to nephrocalcinosis and kidney stones [6]. Moreover, in the absence of H+ secretion in the collecting ducts, potassium excretion is increased, leading to hypokalemia, which is believed to be associated with activating the renin-angiotensin system (RAS) [7].

Acquired RTA is diverse, and its causes may include the exacerbation of chronic diseases. It has been suggested that the worsening of chronic kidney disease (CKD) due to the progression of chronic diseases can lead to RTA [8]. Here, we report a case of a woman in her fifties who presented with RTA, hypokalemia, and hyperuricemia associated with CKD exacerbated by a gout flare. Through this case, we discuss the approach of general practitioners in diagnosing and treating the progression of RTA, hypokalemia, and hyperuricemia in a regional hospital setting.

## Case presentation

A 53-year-old woman visited our hospital with generalized joint pain and difficulty moving. Three years prior (2021), she had been seen at a rheumatology department at a general hospital for generalized joint pain, where she underwent an evaluation for suspected rheumatoid arthritis. Although the rheumatoid factor was positive, hyperuricemia and findings suggestive of tophi in her fingers led to a diagnosis of gouty arthritis. Additionally, she was diagnosed with chronic renal failure, elevated urinary β2-microglobulin, and hypokalemia, consistent with gouty nephropathy. She continued treatment for these conditions, and her joint pain persisted, leading to her referral to our hospital. At our facility, she received outpatient treatment for CKD (stage 4) secondary to hyperuricemia. However, in January of 2024, she stopped attending follow-up visits. In June 2024, she presented again with generalized joint pain and fatigue. Persistent inflammation was noted, and treatment was initiated with prednisolone 20 mg, followed by the addition of daprodustat of 2 mg daily. Her serum potassium levels dropped to 1.9 mEq/L in August of 2024. Although hospitalization was indicated, she refused due to social and economic reasons. Oral potassium chloride (KCL) supplementation was attempted but was ineffective, and her potassium levels decreased further to 1.5 mEq/L. She did not notice any change in the amount of urine per day. As she developed symptoms such as muscle weakness and numbness, and her ability to live independently at home became compromised, hospitalization was eventually deemed necessary.

Her past medical history included emphysema, CKD (stage 5), gastroesophageal reflux disease, and chronic gout. Before admission, her medications included prednisolone 7.5 mg, esomeprazole 20 mg, KCL granules 16 g, daprodustat 6 mg, febuxostat 60 mg, and colchicine 0.25 mg daily.

**Figure 1:**
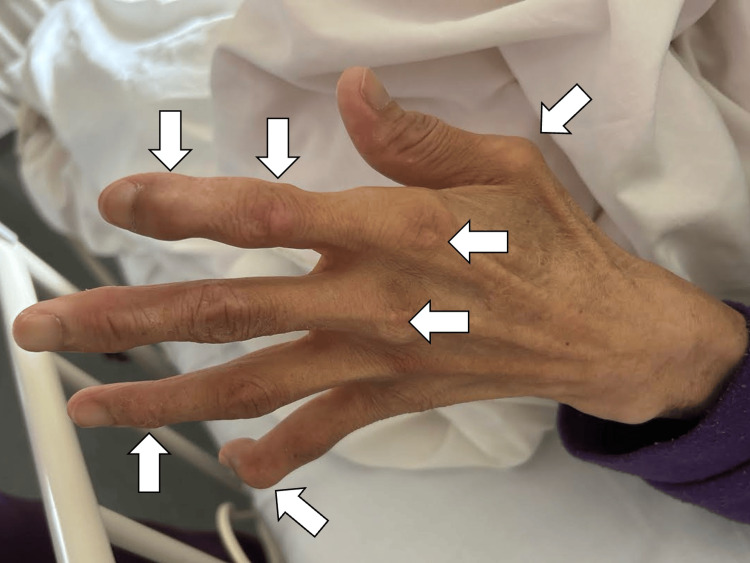
Gouty tophi were observed on her fingers (white arrows).

Upon admission, she was alert and oriented. Her vital signs were as follows: body temperature 36.9°C, blood pressure 97/62 mmHg, pulse rate 84 beats per minute, and oxygen saturation of 98% on room air. Breath sounds were clear, and no abnormal heart murmurs were detected. Gouty tophi were observed on her fingers and the dorsum of her feet (Figure 1).

**Figure 2:**
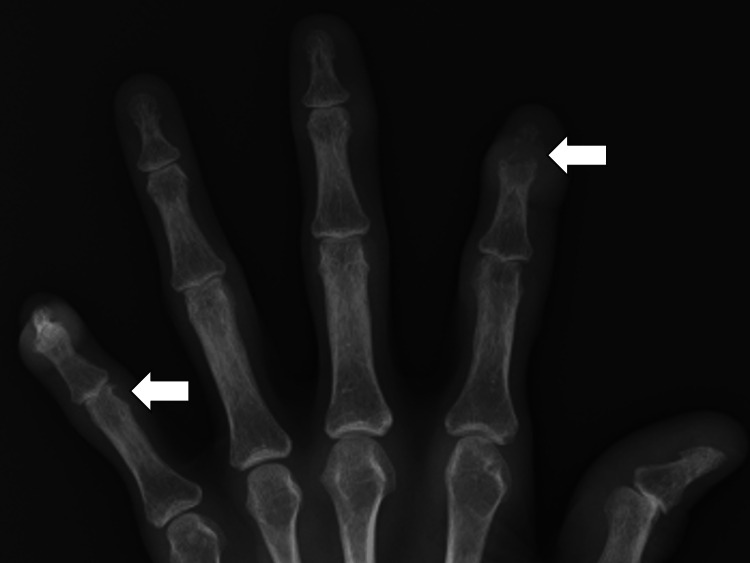
Radiographs of the fingers showing overhanging edges in the joints (white arrows).

Spontaneous pain and tenderness were noted in the proximal interphalangeal (PIP) joints of the second fingers on both hands. Radiographs of the fingers showed overhanging edges in the joints (Figure 2).

Laboratory tests at admission revealed elevated levels of uric acid, creatinine, blood urea nitrogen, C-reactive protein, and erythrocyte sedimentation rate (Table 1).

**Table 1:** Initial laboratory data of the patient CRP, C-reactive protein

Marker	Level	Reference
White blood cells	10.9	3.5–9.1 × 10^3^/μL
Neutrophils	75.5	44.0–72.0%
Lymphocytes	17.8	18.0–59.0%
Hemoglobin	9.4	11.3–15.2 g/dL
Hematocrit	27.5	33.4–44.9%
Mean corpuscular volume	92.2	79.0–100.0 fL
Platelets	52.7	13.0–36.9 × 10^4^/μL
Erythrocyte sedimentation rate	121	2–10 mm/hour
Total protein	6.7	6.5–8.3 g/dL
Albumin	3.2	3.8–5.3 g/dL
Total bilirubin	0.2	0.2–1.2 mg/dL
Aspartate aminotransferase	24	8–38 IU/L
Alanine aminotransferase	21	4–43 IU/L
Lactate dehydrogenase	263	121–245 U/L
Blood urea nitrogen	22.4	8–20 mg/dL
Creatinine	3.53	0.40–1.10 mg/dL
Serum Na	134	135–150 mEq/L
Serum K	1.8	3.5–5.3 mEq/L
Serum Cl	102	98–110 mEq/L
CRP	4.14	<0.30 mg/dL
Urine test	-	-
Leukocyte	-	-
Protein	-	-
Blood	-	-

Arterial blood gas analysis showed a pH of 7.312, pCO_2_ of 34.2 mmHg, pO_2_ of 105.0 mmHg, bicarbonate of 17.3 mmol/L, base excess (BE) of -8.2 mmol/L, sodium of 138 mmol/L, potassium of 2.0 mmol/L, chloride of 108 mmol/L, lactate of 0.8 mmol/L, and an anion gap (AG) of 12.0 mmol/L, indicating hypokalemia and a normal anion gap metabolic acidosis. Urinalysis showed no abnormal findings with a pH of 5.5, a specific gravity of 1.020, red blood cells <1/high-power field (HPF), and white blood cells <1/HPF. Based on these findings, the patient was diagnosed with type 1 RTA, with persistent hypokalemia in the absence of diarrhea and a urinary pH not below 5.5.

Upon admission, she had a fever, but no signs of infection were observed on physical examination, blood tests, or urinalysis. The fever was thought to be due to polyarticular gout, and antibiotics were not administered. By the second hospital day, the fever had subsided, suggesting it was related to systemic inflammation. Regarding her hypokalemia, 120 mEq of potassium was administered over 19 hours on the first day, increasing potassium to 3.5 mEq/L on follow-up arterial blood gas analysis 2 hours later. However, potassium levels dropped to 2.8 mEq/L after 24 hours, and 160 mEq of potassium was administered on the second hospital day. By the third hospital day, potassium levels rose to 5.2 mEq/L, and potassium supplementation was discontinued. On the fourth hospital day, potassium levels decreased to 3.0 mEq/L, and 120 mEq of potassium was administered. In preparation for discharge, oral potassium supplementation was started at 72 mEq/day. The patient's serum potassium levels subsequently stabilized at 5.0 mEq/L, allowing intravenous potassium supplementation to be discontinued. We suggested a renal biopsy to investigate her kidney disease, but she refused the biopsy because of the invasiveness. Her overall condition improved, and thus she was discharged home and scheduled for outpatient follow-up.

## Discussion

This case presents a patient with hyperuricemia, hypokalemia, and CKD, where progressive hypokalemia led to RTA. Chronic urate nephropathy is a type of CKD primarily induced by the deposition of sodium urate crystals in the renal medullary interstitium. The clinical presentation of this condition is often nonspecific, with hyperuricemia being more indicative than other findings, such as proteinuria.

Gouty nephropathy, a well-known chronic renal complication of gout, may have played a significant role in this patient’s condition [9]. Previous studies suggest that urate crystal deposition alone is unlikely to cause end-stage renal disease [10]. However, the traditional notion of gouty nephropathy, characterized by chronic interstitial nephritis due to urate deposition, has been debated [11]. In clinical practice, CKD with a long-standing course is often treated as gouty nephropathy after ruling out other causes of kidney disease, even in the absence of confirmatory renal biopsy [12].

Epidemiologically, studies using health screening data have demonstrated that baseline hyperuricemia is an independent predictor of rapid renal function decline and that a temporal increase in serum uric acid levels contributes to worsening kidney function [13]. A meta-analysis of observational studies on serum uric acid and CKD incidence reported that a 1 mg/dL increase in serum uric acid was associated with a 1.22-fold higher risk of CKD development [14]. This significant association was noted even in individuals younger than 60 years. Similarly, another meta-analysis indicated that a 1 mg/dL increase in serum uric acid was significantly linked to new-onset CKD, with an odds ratio (OR) of 1.15. Hyperuricemia was an independent predictor of CKD development, with an OR of 2.35 [15].

In the present case, serum uric acid levels were poorly controlled. The patient's diet was limited to sweets, resulting in severe malnutrition, with a body mass index (BMI) of 11.4. Effective management of urinary conditions is crucial in preventing the exacerbation of gouty nephropathy and the onset or progression of urolithiasis [16]. Urological management includes reducing urinary uric acid concentration through a low-purine diet, controlling uric acid excretion with uric acid synthesis inhibitors, maintaining adequate urine output, and correcting acidic urine to increase the solubility of urinary uric acid [17]. Patients should be instructed to consume sufficient fluids to maintain a daily urine output of more than 2,000 mL. Dietary management, such as incorporating alkaline foods and using sodium bicarbonate or citrate preparations to correct acidic urine, is recommended to maintain a pH between 6.0 and 7.0 [17]. Although hyperuricemia is common, it can cause kidney damage, leading to advanced CKDs, as in this case. General physicians should be keen on the level of hyperuricemia and manage the condition meticulously.

Chronic hyperuricemia is a common disease and often leads to the deposition of urate crystals in the renal interstitium, whereas acute kidney injury is more frequently associated with the deposition of urate crystals in the renal tubules [17]. In this case, although the patient had chronic hyperuricemia, urate crystal deposition in the renal tubules likely led to the development of RTA, which subsequently resulted in persistent hypokalemia. General physicians must comprehensively control various patients with CKD and hyperuricemia [18]. Although CKD complicated with RTA is rare, general physicians should consider the presence of RTA when patients show unexplained hypokalemia and hyperuricemia.

## Conclusions

This case highlights the chronic presence of hyperuricemia, which likely caused urate crystal deposition in the renal interstitium and tubules, leading to RTA. The resultant acidosis contributed to persistent hypokalemia. Preventive measures against the progression of gouty nephropathy, including a low-purine diet, control of uric acid excretion, and correction of acidic urine, are critical in managing such patients. Tailored dietary and pharmacological interventions are essential for long-term management.
